# Motility in Frail Older Adults: Operationalization of a New Framework and First Insights into Its Relationship with Physical Activity and Life-Space Mobility: An Exploratory Study

**DOI:** 10.3390/ijerph17238814

**Published:** 2020-11-27

**Authors:** Julia Seinsche, Wiebren Zijlstra, Eleftheria Giannouli

**Affiliations:** Institute of Movement & Sport Gerontology, German Sport University Cologne, 50933 Cologne, Germany; jule.seinsche@gmail.com (J.S.); zijlstra@dshs-koeln.de (W.Z.)

**Keywords:** aging, life-space assessment, frailty, mobility determinants

## Abstract

In order to design effective interventions to prevent age-related mobility loss, it is important to identify influencing factors. The concept of “motility” by Kaufmann et al. subdivides such factors into three categories: “access”, “skills”, and “appropriation”. The aim of this study was to assemble appropriate quantitative assessment tools for the assessment of these factors in frail older adults and to get first insights into their relative contribution for life-space and physical activity-related mobility. This is an exploratory cross-sectional study conducted with twenty-eight at least prefrail, retired participants aged 61–94. Life-space mobility was assessed using the “University of Alabama at Birmingham Life-space Assessment” (LSA) and physical activity using the “German Physical Activity Questionnaire” (PAQ50+). Factors from the category “appropriation”, followed by factors from the category “skills” showed the strongest associations with the LSA. Factors from the category “access” best explained the variance for PAQ50+. This study’s findings indicate the importance of accounting for and examining comprehensive models of mobility. The proposed assessment tools need to be explored in more depth in longitudinal studies with larger sample sizes in order to yield more conclusive results about the appropriateness of the motility concept for such purposes.

## 1. Introduction

Mobility can be broadly defined as the ability to move from one place to another with or without mobility aids, or the use of means of transportation [1]. It is important to differentiate between mobility capacity (i.e., what a person “can” do, as measured in laboratory settings) and mobility performance (i.e., real-life mobility, which focuses on what a person actually does in everyday life). Recent evidence showed that mobility capacity does not reflect real-life mobility [2,3,4]. One aspect of real-life mobility relates to physical activity (PA). The domains of physical activity in real life can broadly be classified into occupational physical activity, sports, conditioning, household, and other activities [5]; (these all comprise mobility-related activities such as walking). Further important aspects of a person’s mobility in real life are captured by the so-called life-space mobility (LSM) [6,7]. LSM describes the physical and social environment a person visits in real life. It can also be classified as in- or out-of-home mobility and is defined by the space in which a person moves in daily life (e.g., home, neighborhood, city, or further away). Thus, in accordance with the definition by Webber et al. (2010) [1], LSM encompasses both a person’s independent mobility, requiring mobility-related physical activity (e.g., walking), and all movement supported by mobility aids and/or means of transportation.

Particularly in old age, LSM is very important for social participation [8], but it has also been shown that it is closely connected with a person’s health status, physical functioning [9,10], cognition [11], and quality of life [12]. That is why a restricted LSM is regarded as a red flag for physical disability and even a higher mortality risk [13,14]. The same applies to physical activity, which has been shown to have an effect on physical and psychological health [15,16] and on quality of life [17] and is also negatively associated with a significantly higher risk of premature death [18,19].

In order to develop interventions, which can prevent a decrease of real-life mobility, it is necessary to identify factors that affect it. Several “mobility frameworks” aim at the organization of these factors. A popular one is the framework of Webber et al. (2010) [1], who created a social-economic framework containing five categories; cognitive, psychosocial, physical, environmental, and financial factors. However, Kaufmann et al. (2004) [20] argued that in order to fully understand mobility it is necessary to focus more on the aspirations and plans of people, and on their motivations to be mobile (or immobile). Moreover, previous research has highlighted the importance of environmental factors for LSM. Crucial examples are walkability [21], perception of the environment [22], diversity of neighborhood amenities [23], and access to means of transportation [24]. Kaufmann et al. (2004) [20] developed another framework, the framework of “motility”, which takes these motivation-related and environmental factors into consideration. Motility describes the actual or the potential capacity to be mobile. The influencing factors within this framework are subdivided into three categories:

1. Access (e.g., infrastructure, services and access to facilities and services);
2. Skills (physical and cognitive skills, special competences like a driver’s license);
3. Appropriation (e.g., plans, needs, values and motivations).

Mobility in old age has been addressed by a series of previous studies, which could identify several influencing factors. However, most studies focused on mobility capacity rather than on real-life mobility performance (e.g., [25]). Besides, studies that did focus on real-life mobility have merely analyzed mobility-related physical activity or LSM and motility at a time (e.g., [22,26,27]). All of the few studies [6,28,29,30] that applied a comprehensive mobility framework used the one by Webber et al. (2010). These studies showed that only a small proportion of variance in real-life mobility was explained by the selected multidomain factors. What is more, these studies have assessed either healthy older adults without mobility limitations [6,30], or older adults suffering from severe neurological or cognitive disorders [29]. Kaufmann’s “motility” concept, which can potentially deliver a better insight in mobility (determinants) in old age, has so far only been used in qualitative studies [31,32,33,34], with working-aged adults [35,36] or with non-frail older adults [37,38]. Furthermore, Cuignet et al. (2020) [38] and Bernier et al. (2019) [36] focused on the individual aptitudes and interests regarding different transportation modes excluding for example the general social and psychological contexts. To the best of our knowledge, there are no studies with a quantitative approach assessing multidomain influencing factors based on the ‘motility’ framework in frail older adults. Older adults experiencing frailty are not acutely medically ill but are in a state of compromised function and capacity arising from a reduction in reserve capacity across multiple systems [39]. Therefore, the risk of becoming immobile and suffering from all the negative consequences of restricted mobility is especially high for them, making them a very relevant target group to be monitored over time if the aim is to identify mobility determinants that can be addressed by preventive interventions.

Thus, this pilot study is a first attempt to put together appropriate (age-specific) quantitative assessment tools for the assessments of the three motility factors (access, skills, and appropriation) and an exploration of their relationships with physical activity and life-space mobility. 

The first hypothesis is that factors from all three categories have a significant relationship with LSM of frail older adults, but “appropriation” has the largest. The second hypothesis is that factors from the category skills have the strongest significant relationship with PA, as high physical (and cognitive) functioning (which are components of the category “skills”) are prerequisites to be physically active. 

## 2. Materials and Methods 

### 2.1. Study Design

The study was conducted as an explorative cross-sectional study and is part of a series of studies looking into mobility in old age, which is being carried out at the German Sport University Cologne (Cologne, Germany). The Ethics Commission of this institution examined and approved the present study, which confirms that it complies with the principles of the Helsinki Declaration.

### 2.2. Recruitment and Participants

The recruitment of the study participants took place primarily via senior citizens’ networks and senior gymnastic classes and nursing homes. In addition, posters and flyers were displayed in public places such as supermarkets, doctors’ and physiotherapists’ practices and pharmacies, and an advertisement was placed in a local newspaper. The project was also presented in some senior citizen facilities, offering assisted living, since frail people are often unable to live in their own homes due to difficulties in everyday life.

The inclusion and exclusion criteria were queried by means of a standardized telephone screening or, if necessary, personally on site using the same scheme. Inclusion criteria were the following: (1) age of 60 years or older; (2) retired; (3) ability to walk—either independently or by using assistive devices; (4) good knowledge of German language; and (5) prefrail or frail, defined as reaching at least one of five points on the “FRAIL scale” [40]. Exclusion criteria were: (1) acute injuries or severe illnesses restricting mobility; (2) extraordinary activities that deviate from the participants “usual” mobility patterns (e.g., holidays or visits of grandchildren during the test period; and (3) demential diseases.

All study participants were informed comprehensively about the objectives and the individual tests of the project and signed a declaration of consent regarding their participation and the data privacy policy.

### 2.3. Operationalization 

In order to operationalize the concept of motility for use with frail older adults, a short literature review was conducted to preselect potentially appropriate assessment tools. The main selection criterion was evidence supporting the assumption that the measured functions are related to mobility in older adults. Subsequently, a series of further criteria were: reliability and validity values for use in older adults, feasibility regarding use with frail older people and availability of a validated German version. Finally, for each category (access, skills, and appropriation) a pool of measurement instruments was created (Table 1) in order to test and confirm their feasibility and to filter out the most relevant and most suitable assessments for the use in further similar studies. 

All questionnaires were sent via mail to the study participants directly after their inclusion in the study. The questionnaires had to be filled out at home and returned in person a week later in order to provide the opportunity to ask for further information in case of difficulties of understanding certain items. Subsequently, on the same day, the physical assessments, followed by the cognitive ones, were conducted in the laboratory in the order of their appearance in Table 1. All test procedures, including the explanations, were conducted in a standardized way. As the recruitment of further participants still continued during the start of the assessment period, the exact test dates differed from each other by a few weeks.

### 2.4. Real-Life Mobility Assessment Tools

LSM was assessed using the “University of Alabama at Birmingham (UAB) Life-Space Assessment” (LSA) [62] while the determination of PA was based on the “German Physical Activity Questionnaire 50+” (PAQ50+) [63]. Both questionnaires were sent to each participant via mail with the instruction to be filled out at home and returned together with the rest of the questionnaires described in Section 2.3.

### 2.5. Statistical Analysis

All statistical analyses were performed using SPSS version 26. To get the first insights in the relationships between real-life mobility (LSA and PAQ50+) and the different factors of motility, a correlation analysis was performed using Pearson’s (in the case of normal distribution) and Spearman’s (in the case of not normal distributions) correlation coefficients. Subsequently, Bonferroni–Holm correction was applied to correct for multiple comparisons. After that, stepwise (multiple) regression analyses were performed to assess the predictive power of the categories of motility and demographic factors for the LSA and the PAQ50+. Thus, the latter ones served as dependent variables and the factors of motility as predictors. Before the execution of the regression analyses, the following assumptions were examined: Existence of a linear relationship, normality of residuals, homoscedasticity, and absence of multicollinearity.

Concerning the LSA, the first regression analysis was conducted using the factors, which, continued to be significant after the Bonferroni–Holm correction. To avoid false positive results due to the high number of motility factors with respect to the number of study participants [64,65,66], another regression model was performed using three composite scores equivalent to the three categories of motility. To calculate these composite scores, the data of all significantly correlating factors of motility were transformed to a logarithmic scale (Log10) and averaged under the corresponding category of motility. Finally, in the course of a third regression model, these composite scores were analyzed put together with the significantly correlating demographic factors.

Concerning the PAQ50+, due to the low number of factors significantly correlating just one regression analysis was performed.

The normal distribution of all variables and residuals was determined using the Shapiro–Wilk test. For all statistical tests, the significance level was set to 5%, only for the Shapiro–Wilk test the significance level was 10%.

## 3. Results

In response to the participant recruitment strategies, 55 persons showed interest in participating in the study. On the basis of telephone screenings, 21 persons had to be excluded for not meeting the inclusion criteria. As a result, the measurements were finally conducted with 34 participants. There were four further drop-outs due to difficulties with filling out the questionnaires and another two persons were excluded from the data analysis due to missing data.

### 3.1. Descriptive Statistics

The remaining 28 study participants (18 female) who were included in the data analysis were on average age 79 ± 8 years old. Seven study participants (25%) lived in assisted living facilities and 54% were single. Twenty-four participants (86%) were in a prefrail status according to the FRAIL scale [40] the remaining four study participants (14%) were classified as frail. Furthermore, 86% of the participants were multimorbid. Over the last 12 months, 12 participants (43%) fell at least once and based on a single yes/no question, 15 participants (54%) reported a fear of falling. Fifty-four of them indicated that they were exercising on a regular basis. All of the participants had a driving license and 54% had a car, which they also used. Further descriptive parameters of the sample are presented in Table 2 and Table 3.

### 3.2. Real-Life Mobility (LSA)

In the LSA, the study participants achieved an average of 66.6 ± 24.4 points (min. 16, max. 110 points). Eight participants (28.5%) had a restricted life space (LSA < 60) defined as confined to the neighborhood [62,69]. The average PAQ50+ score was 137.3 ± 90.6 points (min 34.9, max. 398.48 points).

### 3.3. Correlation Analysis

Table 4 illustrates that all categories of motility had at least one significant association with the LSA. Thereby, just 1 out of 10 factors of the category access had a significant relationship with the LSA, while skills had 6 (out of 9) significant factors and appropriation 7 out of 13. With regard to the PAQ50+, just two categories showed significant associations: access and appropriation, whereas both categories included just one significant factor.

After the Bonferroni–Holm correction, four factors still significantly correlated with the LSA (Lubben scale and UCLA loneliness scale from the category appropriation and HOTAP and the computer literacy scale (CLS) B-subscale from the category skills) and just one with the PAQ50+ (NEWS-G from the category access).

Regarding the demographic factors, only age (r = −0.424 *) and frailty (r = −0.538 **) demonstrated significant relationships with the LSA, whereas gender and years of education showed none. The PAQ50+ and demographic factors had no significant relationships and neither did the PAQ50+ und the LSA among themselves.

### 3.4. Regression Analysis LSA

A screening of the results with regard to a possible multicollinearity of the determination factors revealed variance inflation factor (VIF) values between 1.0 and 1.036, which did not indicate multicollinearity. All regression models had a high goodness of fit (corrected R^2^ = 0.614, R^2^ = 0.360 and R^2^ = 0.360; Cohen, 1988), with the first one explaining the variance of LSA to the greatest extent. 

No factors from the category access remained significant in any of the regression models. However, factors from the categories skills and appropriation not only showed strong associations with the LSA according to the first regression model, but also appear in the second and third regression model. Thereby, social factors (Lubben Scale) and executive skills (HOTAP) stood out in particular.

The results of the third regression model, including demographic factors (age and TFI) in the analysis of the composite scores, were completely identical with the results of the second regression analysis, which underlined the strong associations of the categories skills and appropriation with the LSA (Table 5).

### 3.5. Regression Analysis PAQ-50+

As according to the correlation analysis only two factors correlated significantly with the PAQ50+, just one regression analysis was conducted. The analysis’ VIF value was 1.0, which did not indicate multicollinearity and the coefficient of determination showed a moderate goodness of fit (R^2^ = 0.199). The category access revealed the biggest association with the PAQ50+ and the fact that access was only represented by NEWS-G highlights the importance of traffic safety as an important environmental factor for PA. Neither the category skills nor the category appropriation showed significant relationships with the PAQ50+ (Table 6).

## 4. Discussion

To our best knowledge, this pilot study is the first one to investigate both life-space mobility and physical activity, and their determinants in frail, older people using the concept of motility as a framework. Aims of the study were to operationalize the concept of motility for quantitative studies in older adults and to analyze the relationships of the three categories of motility (access, skills, and appropriation) with real-life mobility of frail older people. The LSA and the PAQ50+ served as assessments for LSM and PA. In addition, a high number of age-specific performance-based tests and questionnaires were selected and used in order to operationalize the concept of motility and served as predictors of LSA and PAQ50+. So far, just four studies examined a comparable range of possible determination factors [6,28,29,30]. Regarding LSM, the results indicate an association of appropriation followed by skills; however, an association of access could not be proven. PA, on the other hand, was exclusively associated with the category access, while skills and appropriation showed no associations at all. Consequently, both hypotheses (that all categories would have a significant relationship with LSM with appropriation having the biggest/greatest one and that skills would have the largest relationship with PA) need to be rejected.

### 4.1. Participants

This study aimed to examine older adults with compromised physical functioning and thus in risk of restricted mobility performance. According to the FRAIL scale, which also served as an inclusion criterion, most of the participants (86%) were classified as prefrail and the same amount were multimorbid. Their low functional capacity is also evident by the average score at the Tilburg frailty indicator (=5.3, with a score >5 indicating frailty) and the average TUG score (9.1 ± 2.9) [70]. With an average score of 66.6 ± 24.4 points on the LSA, our sample’s life-space mobility is comparable to previous studies with prefrail/frail older adults [13]. The standard deviation of the LSA scores is comparable to other similar studies as well [13,62,71]. Nevertheless, it is rather high and together with the large total range of values it presents a high variability of the participants’ LSA. The same applies to the PAQ50+ scores. However, this is explicable, since some of the study participants lived in assisted living facilities while others were community-dwelling and in some cases even house owners.

### 4.2. Operationalization of the Motility Framework

Kaufmann’s “motility” framework was operationalized using assessment tools specifically validated for use in older adults. The operationalization strategy was based on our previous work [6] and an additional literature review aiming to find determinants of real-life mobility (especially for older adults) and afterwards on creating a large pool of assessment tools meeting the inclusion criteria. Apart from two assessments (EAMQ and SAQ, for which own translations, verified by back translation were used for this study) all other questionnaires were already available in the German language and all tests showed acceptable to good psychometric qualities. Due to the exploratory approach, the final selection included a high number of tests (access: 10; skills: 8; appropriation: 13) in order to get sufficient insights into the topic and to assess the instruments’ feasibility in the use with frail older people. Indeed, some assessment told seemed to be more appropriate than others. The assessments used to assess the category “access” were feasible with this study sample. Despite the lack of significant associations with real-life mobility in the present study, other studies prove that their inclusion is vital in further studies looking deeper in its association with real-life mobility [72,73]. There were also no issues with any of the performance-based physical and cognitive tests, which were used to cover the category “skills”. However, MDPQ overstrained the participants and we suggest to only use the subscales relevant for real-life mobility such as the subscale “communication” and “internet”. The “appropriation” related assessment tools showed a lot of significant correlations with LSA but still, they were very time-consuming, which raised some complaints by the participants. Further studies with similar samples can reduce for example the number of self-efficacy scales (FES, ABC-D, and mGES) as all of them cover similar aspects and yielded comparable results.

### 4.3. Access

Environmental factors are being increasingly taken into account in mobility research. Previous studies showed the influence of a series of different environmental features on LSM—either facilitating or restricting it. Examples of mobility barriers are a poor infrastructure, uneven sidewalks, loud traffic, the absence of a possibility to take a break during walking, and long ways to various service facilities [74,75]. Mobility facilitators are green spaces [76], pavements, the attractiveness of the neighborhood [22], and the variety of amenities [23]. However, in the present study, no associations between any environmental factors and LSM could be found. This might be due to the low variability of the study participants’ living environments, as they all lived in urban areas, very similar to each other.

Contrary to LSM, environmental factors did seem to play a role for physical activity, as one factor from the category “access” (traffic-safety), showed significant relationships with the PAQ50+score even after applying the Bonferroni–Holm correction and also by the results of the regression analysis. This is partly in line with other studies, which found that unsafe or uneven footpaths with a high tripping risk, a lot of traffic and in general traffic hazards are barriers for PA, especially in older people [77,78,79,80,81]. It has been suggested that older adults are more sensitive to environmental obstacles and facilitators due to a decreased self-efficacy when it comes to unsafe roads, street crossings or missing sidewalks and because they spend more time in the neighborhood compared to working people [82]. Moreover, low traffic safety is often connected to a high traffic volume, which in turn leads to air pollution and subsequently to a lower motivation to be physically active outside. Of course, this applies primarily to walking (either leisure walking or transportation walking) and not to domestic PA. One reason why no further associations could be shown in the present study might be, as Strath et al. (2012) [83] pointed out, that many objectively measured environmental factors show a much stronger impact on PA than self-perceived ones. On the other hand, according to Kirtland et al. (2003) [84] the difference between perceived and objective environmental measures in their association with PA, respectively the accuracy of people’s perception, is among others based on the people’s level of physical activity. Besides it is depending on the environmental factor under investigation. They found that people who are insufficiently active have the highest associations with perceived safety. This again could be an explanation for the perceived “traffic safety” having an association with PA in the present study analyzing frail older adults who are rather inactive while other environmental factors might be more accurately measurable with objective assessments. Finally, when analyzing access-related factors it has to be considered that selective migration could be a potential bias, because people may select neighborhoods based on their physical abilities instead of allowing the environment to influence their physical activity behavior. On the other hand, older people are probably not that likely to move to another neighborhood compared to younger people who have not settled down yet. 

### 4.4. Skills 

All three regression models showed a significant relationship between factors from the category “skills” and LSM. The HOTAP test stood out in particular since it was still significantly associated with LSM after the Bonferroni–Holm correction and it also remained as a significant factor in the regression model. The HOTAP test is a measure of executive functions, which, according to other studies, are of great importance for a successful navigation through unknown spaces, because they include the ability to plan and organize and have decision making capacity [85]. Basically, all studies analyzing such a connection report on positive association between executive functions and life-space mobility [4,85,86,87]. Contrary to this, the results of studies analyzing the effect of other cognitive functions such as global cognition, memory, and processing speed are varying (e.g., [29,30,85,87,88]). One explanation is that some of those studies included persons with severe cognitive impairments and/or used assessments like the MMSE to measure global cognition [29,62,87], which is not a tool to measure cognitive performance but a screening tool for the assessment and grading of cognitive impairments [89]. That is why even in this prefrail/frail sample it showed considerable ceiling effects and cannot serve as a comparison instrument. However, in general, studies do agree that decreased cognitive skills impede moving in larger outdoor spaces. In addition, studies like De Silva et al. (2019) [90] also describe an opposite effect stating that a reduced, homogenous life-space without special cognitive, auditory, or visual stimuli causes a decline in cognitive abilities with one LSA score of 41 points seemingly being the cut-off value leading to a steeper decline of cognition.

However, contrary to LSM, the current study’s findings did not show any significant associations of cognitive skills with PA. This may indicate that for this study’s participants, moving in life-spaces further away from home posed a greater cognitive challenge than PA in well-known areas closer to their homes.

According to all regression models, none of the physical skills is a significant determinant for real-life mobility. This applies to LSM and to PA and is not in line with most other studies showing a positive impact of physical skills like muscle strength and various gait parameters on LSM [29,62,91,92] and on PA [93,94]. However, the study samples had a higher minimum age [91], dementia [92], or used objective data for the measurement of real-life mobility. On the other hand, relating to life-space mobility, Harada et al. (2017) [95] and Blamoutier et al. (2017) [96] support the findings of this study as they showed a relationship between hand grip strength and mobility capacity but not with LSM—neither when measured with the help of objective assessment (GPS data) nor with subjective assessment (LSA) [96]. The latter findings confirm the conclusions of Giannouli et al. (2016) [3] showing that mobility capacity measured in a laboratory does not correspond to real-life mobility.

Furthermore, even some form of PA appeared to be possible despite physical limitations. One reason might be that the PAQ50+ includes activities that do not require huge physical abilities (e.g., light household activities and cooking). So, PA defined as any bodily movement seemed to be rather independent of physical skills. Certainly, it has to be considered that this probably differs depending on the level of frailty. A frailer sample may at some point be unable to compensate for their physical disabilities. Blamoutier et al. (2017) [96] indicate something similar as well.

Still, these results support our general idea that, unlike common expectations, real-life mobility is determined by more factors than simply physical skills. Apparently even frail participants seemed to have found other strategies to compensate for their physical limitations and to be mobile inside and outside. This is supported by the fact that no significant correlation between PAQ50+ and LSA themselves could be detected, showing that the study participants could be very active in small life-spaces or were able to reach areas further away from home despite decreased physical abilities—probably using aids or means of transportation.

The technology-related competences did not show significant associations with real-life mobility either, which could mean that the participants used other aids like newspapers or maps to organize their social life and to navigate through unknown areas. It could be taken into consideration that at least in the correlation analysis, the CLS questionnaire showed a strong correlation with life-space mobility, which is further emphasized by the results of the Bonferroni–Holm method. As part B more reflects theoretical knowledge about computer related topics than actual user experience, it can be assumed that the results also mirror the participants’ cognition and probably their age as well instead of simply their use of computers as a mean for navigation.

All in all, unlike our expectations, the category skills showed no associations with PA, but instead, it did have significant relationships with LSM. Future studies should further investigate the relative contribution of physical and cognitive functions for physical activity in frail older adults.

### 4.5. Appropriation

We found a significant association of the category “appropriation” with LSM. Thereby, social factors seemed to play a special role, since Lubben’s social network scale remained as a significant predictor in the regression model. This is in accordance with previous studies [29,97]. In fact, social activities have been found to be the reason for a large proportion (about 20%) of trips outside of home [98]. With regard to social life, neighbors play a decisive role, as older people who maintain good contact with their neighbors turned out to have a better walking activity and move further away from home [99,100]. This is because neighbors can serve as walking companions [101] and therefore as a motivating factor to be physically active. Surprisingly, factors like spatial anxiety, rigidity, and even balance and gait related factors did not have significant relationships with LSM. This is perfectly in line with Ullrich et al. (2019) [29], but contradicts many other studies [27,93,102]. One reason for this might be that in contrast to the other studies, we (just like Ullrich et al. (2019) [29]) analyzed a wide range of potential predictors of mobility. So, accounting for all the other factors reduces the importance of these psychological determinants.

Based on the results showing a strong relationship between social factors and LSM, it could be assumed that social life had a big impact on PA as well—an assumption, which is also supported by other studies [99,103,104,105]. However, in the present study no relationship between the category appropriation and PA could be shown. One explanation might again be that in many studies PA has been defined as walking activity, which also explains the common outcomes of studies analyzing the impact of social factors on PA or on LSM. Yet, as already mentioned, the present study included recreational and household activities as well, which are assumingly not primarily affected by social connections. Moreover, simpler activities performed at home (providing a safe environment) do not require such a high feeling of self-efficacy.

Still, in the present study and in general, “appropriation” is strongly associated with life-space mobility, because appropriation related factors like social life provide a stimulus, support, and a feeling of safety for the real-life mobility of frail older people—especially when moving outside [104,106]. 

### 4.6. Limitations

An important limitation of this study is its cross-sectional design, which does not allow causal implications. A longitudinal study monitoring mobility and motility of older persons’ over time would be needed to confirm the impact of any changes in the motility categories on real-life mobility. Still, the present study provides a good overview of possible age-appropriate assessments and validates the motility framework for the use in older adults. Another limitation was the small sample size, which combined with the large amounts of assessment tools, might have led to false positives in the correlation analysis results. However, to correct for multiple testing we applied the strict Bonferroni–Holm method and also built composite scores prior to performing the regression analysis, making its results free of bias. Still, the difficulties in recruiting a larger sample size, which fitted the inclusion criteria of this study (mainly the frailty criterion) highlights the barriers in accessing such samples, who are actually the most relevant to investigate. Further studies should look into strategies to recruit frail older adults. Finally, the study did not control for poor nutrition/malnutrition, which is known to have an influence on frailty and thus could also play a role in older adult’s real-life mobility. This is another aspect future studies should take into consideration.

## 5. Conclusions

The results show that to a certain extent all three categories of motility had significant associations with real-life mobility. Concerning the category access, the results showed a strong significant relationship of “safety from traffic” with PA, but none with LSM. The category “skills” had the second biggest association with LSM with the executive functions standing out in particular. Still, the weak correlations of physical factors showed that compared to appropriation, limited skills could be compensated more easily. For example, walking difficulties may be compensated by slower walking speed, alternative routes, or the use of means of transport. That is probably why psychosocial factors summarized under appropriation show the strongest relationship with LSM with especially social networks proven to be important and comprehensive determinants. However, skills and appropriation did not have any significant associations with PA.

To sum up, appropriation followed by skills best explain the variance in LSM and the category access the variance in PA of frail older people. Finally, our results underline that real-life mobility is a very complex, multidimensional construct, and therefore the concept of motility is an appropriate tool for its analysis as it integrates a high number of multidimensional factors.

## Figures and Tables

**Table 1:** Assessment tools.

Assessment Tool	Outcome	Description
**Access**		
NEWS ^1^, A Score [41]	Types of residences in the neighborhood	Questions about the amount of different types of houses in the neighborhood like single houses, townhouses, and apartment houses.
NEWS, B Score	Availability of stores and other facilities in the neighborhood	Questions about duration to get to the nearest stores or facilities (e.g., supermarkets, post office, library, restaurants, banks, fitness center, or pharmacies).
NEWS, C Score	Access to services	Questions about the accessibility of services or means of transport. Its focus is on the possibilities to reach services on foot.
NEWS, D Score	Types of streets in the neighborhood	Questions about the neighborhood’s streets characteristics like cul-de-sacs, footpaths or (multilane) street crossings.
NEWS, E Score	Availability of places for walking and cycling	Questions about the neighborhood’s sidewalks and, if available, cycle tracks.
NEWS, F Score	Neighborhood attractiveness	Questions about the availability of trees, nature, and beautiful houses in the neighborhood.
NEWS, G Score	Traffic safety	Questions about the amount of traffic, traffic speed, air pollution, and the effect of traffic on the quality of walking activities.
NEWS, H Score	Crime safety	Questions about street lighting, crime rate, and how bustling the neighborhood is.
NEWS, I Score	Neighborhood satisfaction	Questions about satisfaction concerning different environmental topics like walkability, access, noise, services, and social life.
Environmental Analysis of Mobility Questionnaire (EAMQ) [42]	Mobility obstacles caused by the environment	Questions about environment-related obstacles and their handling. Twenty-four situations (e.g., crossing heavily frequented roads, walking in darkness, visiting unknown or crowded places) are described and each situation is considered with two questions: How often the participants are confronted with these situations and how often they avoid them.
**Skills**		
Maximum Hand Grip Strength (HGS; kg; Jamar Hand dynamometer)		The task is to sit up with the forearms on the armrest and the wrist over the edge. The dynamometer is held with the stronger hand and must be pushed as strongly as possible.
Five Times Sit to Stand (5×StS) [43]	Leg power	The test starts with the participant sitting on a chair without armrests. Outcome parameter is the time needed to stand up five times as quickly as possible.
Timed Up-And-Go Test (TUG), 3-meter version [44]	Functional mobility	The task is to get up from a chair, walk three meters straight ahead, turn around, walk back to the chair, and sit down again.
Trail-Making-Test A (TMT A) [45,46]	Psychomotor speed and Cognitive flexibility Processing speed	Numbers (1–25) written down on a paper in a randomized way have to be connected in an ascending order without lifting the pen from the paper. Time to complete the tasks is evaluated.
Trail-Making-Test B (TMT B) [45,46]		Numbers and letters have to be connected alternately in an ascending order. Time to complete the task is evaluated.
Handlungsorganisation und Tagesplanung (HOTAP) [47]	Planning (Executive functions)	Single steps of eight different everyday activities/tasks (e.g., making coffee or lawn mowing) are pictured on several different cards, which must be put into the right order. Time and the errors at the sequence’s order are evaluated.
Computer Literacy Scale, B-subscale (CLS B) [48]	Computer literacy	Questions about computer-related symbols and functions.
Mobile Device Proficiency Questionnaire (MDPQ) [49]	Ability to handle mobile devices	Questions related to the use of mobile devices for different purposes (e.g., communication, internet browsing etc.)
**Appropriation**		
Falls Efficacy Scale—International Version (FES-I) [50]	Fear of Falling	Questions about the participant’s concerns regarding falls during different everyday activities (e.g., stair climbing, cooking, walking on uneven grounds).
Activities-Specific Balance Confidence Scale—German Version (ABC-D) [51]	Balance-associated self-efficacy	Questions about the participant’s subjective evaluation concerning his/her balance during different everyday activities
Modified Gait Efficacy Scale (mGES-D) [52]	Gait-related self-efficacy	Questions about walking-related self-efficacy in different environmental conditions or situations (e.g., walking on grass, climbing stairs with or without an available handrail)
University of California, Los Angeles, Loneliness Scale (UCLA Loneliness Scale) [53]	Loneliness and social isolation	Questions like “How much of the time do you feel that you are “in tune” with the people around you?” are used to assess loneliness
Multidimensional Personality Test for Adults 1 (MPTE-1) [54]	Arousal	Question like “When I haven’t done anything for a couple of days, I become restless”
Multidimensional Personality Test for Adults 2 (MPTE-2) [54]	Rigidity	Questions like “I am reluctant to give up certain habits, because I get attached to them easily”
Spatial Anxiety Questionnaire (SAQ) [55]	Spatial Anxiety	Questions about the participant’s anxiety during navigation in unknown areas like for example in order to find the way to an appointment
Short Form 12 (SF12) physical health status [56,57]	Quality of life	The questionnaire is a short form of “SF-36” and asks for the participant’s health-related (physical and psychological) quality of life.
Short Form 12 (SF 12) psychological health status		
Lubben Scale [58]	Social networks	Questions like “How many relatives do you see or hear from at least once a month?” are about a person’s social networks with the focus being on family and friends.
Ageism Survey (Palmore Scale) [59]	Perceived Ageism	The questionnaire confronts the participant with twenty different occasions/situations, which could be examples for ageism. An exemplary question is “I was ignored or not taken seriously because of my age”.
Tangible Support Subscale (TSS) [60]	Perceived Help Availability	It asks for perceived help availability with regard to tasks like repairing the car, giving a ride to a doctor or the airport or borrow money.
Data entry form of the social situation (SoS) [61]	Social status	It includes four dimensions “social contacts and support, social activities and the living and economic situation”

^1^ NEWS: Neighborhood Environmental Walkability Scale.

**Table 2:** Descriptive statistics of the demographic factors.

Demographic Factors	Mean ± SD ^1^	Min. ^2^	Max. ^3^
Age	78.7 ± 7.9	61	94
BMI	25.9 ± 4.9	18	38.7
Number of chronic diseases	5.3 ± 3.1	0	13
Years of education	12.5 ± 5.4	3	26
Tilburg Frailty Indicator (TFI) [67]	5.8 ± 2.9	1	11
Mini-Mental State Examination (MMSE) [68]	28.9 ± 0.9	26	30

^1^ SD: standard deviation; ^2^ Min: Minimum; ^3^ Max: Maximum.

**Table 3:** Descriptive statistics of the factors of motility.

Assessment Tools	Mean ± SD ^1^
**Access**	
NEWS ^2^, A Score [41]	252.6 ± 45.6
NEWS, B Score	3.0 ± 0.7
NEWS, C Score	3.4 ± 0.7
NEWS, D Score	2.8 ± 0.4
NEWS, E Score	2.9 ± 0.6
NEWS, F Score	2.9 ± 0.5
NEWS, G Score	2.6 ± 0.6
NEWS, H Score	3.1 ± 0.5
NEWS, I Score	2.1 ± 0.5
EAMQ ^3^ [42]	13.3 ± 6.9
**Skills**	
Maximum Hand Grip Strength (HGS; kg; Jamar Handdynamometer)	16.5 ± 8.3
Five Times Sit to Stand (5×StS) [43]	12.3 ± 2.9
Timed Up-And-Go Test (TUG), 3-meter version [44]	9.1 ± 2.9
Handlungsorganisation und Tagesplanung (HOTAP) [47]	10.4 ± 5.3
Trail-Making-Test A (TMT A) [45]	42.7 ± 12.5
Trail-Making-Test B (TMT B) [45]	121.4 ± 52.9
Computer Literacy Scale, B-subscale (CLS B) [48]	14.5 ± 8.4
Mobile Device Proficiency Questionnaire (MDPQ) [49]	15.9 ± 9.3
**Appropriation**	
Falls Efficacy Scale—International Version (FES-I) [50]	21.7 ± 3.8
Activities-Specific Balance Confidence Scale—Deutsch (ABC-D) [51]	1290.1 ± 307.3
Modified Gait Efficacy Scale (mGES) [52]	79.9 ± 19.6
University of Carolina, Los Angeles, Loneliness Scale (UCLA Loneliness Scale) [53]	36.8 ± 11.2
Mehrdimensionaler Persönlichkeitstest für Erwachsene 1 (MPTE-1) [54]	33.1 ± 6.1
Mehrdimensionaler Persönlichkeitstest für Erwachsene 2 (MPTE-2) [54]	37.4 ± 5.7
Spatial Anxiety Questionnaire (SAQ) [55]	10.9 ± 7.3
Short Form 12 (SF12) physical health status [56,57]	36.5 ± 11.9
Short Form 12 (SF 12) psychological health status [56,57]	37.7 ± 12.2
Lubben Scale [58]	28.2 ± 11.6
Ageism Survey (Palmore Scale) [59]	4.0 ± 4.1
Tangible Support Subscale (TSS) [60]	22.3 ± 5.5
Erhebungsbogen der sozialen Situation (SoS) [61]	21.4 ± 2.2

^1^ SD: standard deviation; ^2^ NEWS: Neighborhood Environmental Walkability Scale; ^3^ EAMQ: Environmental Analysis of Mobility Questionnaire.

**Table 4:** Relationship of the factors of motility with life-space mobility and physical activity (Spearman’s/Pearson’s coefficient of correlation, r (* *p* < 0.05; ** *p* < 0.01)).

Category	Factors	Life−Space Mobility (LSA) ^1^	Physical Activity (PAQ50+) ^2^
**Access**	NEWS, A Score	−0.164	0.033
	NEWS, B Score	−0.263	−0.214
	NEWS, C Score	0.029	−0.283
	NEWS, D Score	0.115	−0.065
	NEWS, E Score	−0.133	−0.104
	NEWS, F Score	0.162	−0.125
	NEWS, G Score	−0.07	−0.422 *
	NEWS, H Score	0.221	−0.097
	NEWS, I Score	−0.476 **	0.177
	EAMQ Total Score	−0.267	−0.059
**Skills**	HGS	0.413 *	0.230
	5×StS	−0.415 *	−0.252
	TUG	−0.452 **	−0.309
	HOTAP	0.545 **	−0.041
	TMT A	−0.286	−0.163
	TMT B	−0.441 **	−0.101
	CLS B−subscale	0.506 **	−0.037
	MDPQ	0.007	0.186
**Appropriation**	FES−I	−0.463 **	0.149
	ABC−D	0.397 *	0.068
	mGES	0.436 *	0.060
	UCLA Loneliness Scale	−0.561 **	0.195
	MPTE−1	−0.326 *	0.355 *
	MPTE−2	−0.135	0.236
	SAQ	−0.092	0.098
	SF12 physical health status	0.156	0.089
	SF 12 psychological health status	0.275	−0.136
	Lubben Scale	0.649 **	0.002
	Ageism Survey (Palmore Scale)	−0.036	0.210
	TSS	0.284	0.208
	SoS (Nikolaus)	0.363 *	0.015

^1^ LSA: Life-Space Assessment; ^2^ PAQ50+: Physical Activity Questionnaire 50+.

**Table 5:** Regression models LSA.

Regression Model	Predictor	Beta ^1^	Corrected R^2 2^
Regression model: stepwise regression analysis of the factors still significant after Bonferroni-Holm correction (Lubben scale, HOTAP, UCLA Loneliness Scale, CLS B-subscale)	Lubben Scale (social networks)	0.643 **	0.614 **
	HOTAP (Organizing activity and planning of daily life)	0.471 **	
2. Regression model: Composite Scores (Access, Skills, Appropriation)	Appropriation Score	0.571 **	0.360 **
	Skills Score	0.411 *	

^1^ Beta: standardized coefficient of regression of the predictors of life-space mobility; ^2^ Corrected R^2^: Coefficient of determination (* *p* < 0.05 and ** *p* < 0.01).

**Table 6:** Regression models PAQ-50+.

Regression Model	Predictor	Beta ^1^	Corrected R^2 2^
Regression model: Stepwise regression analysis of all significantly correlating factors	NEWS-G (traffic safety)	−0.478 *	0.199 *

^1^ Beta: standardized coefficient of regression of the predictors of life-space mobility; ^2^ Corrected R^2^: Coefficient of determination (* *p* < 0.05).
